# Screening of key biomarkers of tendinopathy based on bioinformatics and machine learning algorithms

**DOI:** 10.1371/journal.pone.0259475

**Published:** 2021-10-29

**Authors:** Ya xi Zhu, Jia qiang Huang, Yu yang Ming, Zhao Zhuang, Hong Xia

**Affiliations:** 1 District 1, Department of Orthopedics, Xiangtan Central Hospital, Yuhu District, Xiangtan City, Hunan Province, China; 2 Nanhua University, Hengyang City, Hunan Province, China; 3 Department of Orthopedics, Xiangtan Central Hospital, Yuhu District, Xiangtan City, Hunan Province, China; 4 Academy of Anesthesiology, Weifang Medical University, Weifang, China; University of Science and Technology Liaoning, CHINA

## Abstract

Tendinopathy is a complex multifaceted tendinopathy often associated with overuse and with its high prevalence resulting in significant health care costs. At present, the pathogenesis and effective treatment of tendinopathy are still not sufficiently elucidated. The purpose of this research is to intensely explore the genes, functional pathways, and immune infiltration characteristics of the occurrence and development of tendinopathy. The gene expression profile of GSE106292, GSE26051 and GSE167226 are downloaded from GEO (NCBI comprehensive gene expression database) and analyzed by WGCNA software bag using R software, GSE26051, GSE167226 data set is combined to screen the differential gene analysis. We subsequently performed gene enrichment analysis of Gene Ontology (GO) and "Kyoto Encyclopedia of Genes and Genomes" (KEGG), and immune cell infiltration analysis. By constructing the LASSO regression model, Support vector machine (SVM-REF) and Gaussian mixture model (GMMs) algorithms are used to screen, to identify early diagnostic genes. We have obtained a total of 171 DEGs through WGCNA analysis and differentially expressed genes (DEGs) screening. By GO and KEGG enrichment analysis, it is found that these dysregulated genes were related to mTOR, HIF-1, MAPK, NF-κB and VEGF signaling pathways. Immune infiltration analysis showed that M1 macrophages, activated mast cells and activated NK cells had infiltration significance. After analysis of THE LASSO SVM-REF and GMMs algorithms, we found that the gene *MACROD1* may be a gene for early diagnosis. We identified the potential of tendon disease early diagnosis way and immune gene regulation *MACROD1* key infiltration characteristics based on comprehensive bioinformatics analysis. These hub genes and functional pathways may as early biomarkers of tendon injuries and molecular therapy level target is used to guide drug and basic research.

## Background

Tendinopathy is usually described as pathological changes in injured and diseased tendons, which in turn lead to limb pain and functional decline. It is characterized by abnormalities in the molecular structure, composition and cell matrix of the tendon [1]. In recent years, the prevalence of tendinopathy has gradually increased, and some tendinopathy patients have long-term or permanent limb dysfunction and loss [2]. Tendinopathy is more common in limbs and accounts for 30%-50% of muscle skeletal system and locomotor system diseases [3,4]. It is well known that the causes of tendinopathy include internal and external factors. External factors mainly cause acute tendon injury, while chronic tendinopathy is the result of both external and internal factors [5]. Previous studies have suggested that the imbalance of normal homeostasis in tendon tissue, including immune cell infiltration, stromal cell dysfunction, cell apoptosis, oxidative stress, and stromal dysfunction, and these comprehensive conjunct factors lead to the early pathological changes of tendon [1]. Unfortunately, the current diagnosis of tendinopathy has reached the middle and late stage, but the treatment plans are mostly targeted at the later stage of the disease, including centrifugal exercise [6,7] drug injection and surgical treatment [8], etc. Studies have shown that the efficacy and evidence of most treatment plans for tendinopathy are still insufficient. Therefore, early diagnosis and treatment are the key to complete recovery of the disease. At present, the hub genes and pathways in the early stage of tendinopathy are blank studied, and understanding the key pathways that affect the regulation and dynamic homeostasis of the extracellular matrix in the early stage is critical for future targeted therapies of tendinopathy. Therefore, more in-depth studies are urgently needed to elucidate the hub genes.

With the development of high-throughput omics, bioinformatics analysis has become an important tool for identifying potentially hub genes and signaling pathways in a variety of diseases [9,10]. At present, the research progress of bioinformatics analysis of tendinopathy gene expression has not been insufficient, and only a small amount of lncRNA and mRNA related to tendinopathy has been studied. Among them, studies have analyzed the related characteristics of lncRNAs and mRNAs in rotator cuff tendinopathy, including NONHSAT209114.1, ENST00000577806, NONHSAT168464.1, PLK2, TMEM214 and IGF2 [11,12]. Unfortunately, the use of bioinformatics to analyze DEGs related to tendinopathy still needs further research. In this study, we downloaded the original data from the NCBI Gene Expression Synthesis Database (GEO) and constructed a co-expression network through the data set GSE106292 and determined the correlation gene module. By processing the data set GSE26051 and GSE167226 carry on incorporative and differential gene screening. We take the intersection of differential genes and gene modules to obtain common differential genes, and then performed gene Ontology (GO) and Kyoto Encyclopedia of Genes and Genomes (KEGG) enrichment analysis. Then, we used the whole gene expression data of tendon tissues for immune cell infiltration analysis. By establishing LASSO regression model [13], Machine learning algorithm Support vector Machine (SVM-REF) [14], Clustering algorithm Gaussian Mixture model(GMMs) [15], Screening of genes for early diagnosis. The objective of this study was to identify hub genes and pathways that contribute to the occurrence and development of tendinopathy at the molecular level, and to identify candidate genes for early diagnosis and treatment targets.

## Materials and method

### Data collection and download

GEO (Gene Expression Omnibus) database (http://www.ncbi.nlm.nih.gov/geo/) is an international public database, used to store and provide free microarray, second-generation sequencing and high-throughput functional genome data sets [16]. We searched and downloaded the GSE106292, the GSE26051, the GSE167226 data set using the R software GEO database [17–19]. The GSE106292 data sets included the gene expression profiles of 35 cases of tendon, bone, muscle, cartilage and ligament. The GSE26051 data sets included gene expression profiles from 23 patients with chronic tendonopathy and 23 normal tendons; the GSE167226 data sets included gene expression profiles from 19 patients with tendonopathy. Institutional Review Board approval was not required because the study was based on a public database and did not involve animal or human studies.

### Data preprocessing and DEG screening

The original data of GSE106292, GSE26051 and GSE167226 datasets were corrected and normalized by using log2 of the R software. Bioconductor platform (http://www.bioconductor.org/) gene comments file is used to probe the matrix [20]. At the same time, the expression matrix of GSE26051 and GSE167226 datasets was merged, and the differences between batches were eliminated using the Limma software package. The differences of gene expression profiles between GSE26051 and GSE167226 datasets were analyzed by using the Limma function and the Remove Batch Effect function using Limma [21]. By deleting the genes with too low expression value, the expression profile value was converted to log2-counts per million (logCPM), and linear regression was performed to construct the comparison matrix. Where each row represents the gene name and each column represents the sample name of this study. Based on Bayesian calculation of T-values, F-values and log-odds, the eligible differential genes were screened for |log2(FC)| > 1 and PValue < 0.05, and the data was visualized by plotting volcano plots using the ggplot2 program package [22].

### Gene WGCNA analysis

The expression matrix of GSE106292 data sets was selected to construct the expression profile of potential related genes in tendinopathy. The WGCNA software package is an open source and widely used method for identifying co-expression networks in R software [23]. The Pearson correlation coefficient is calculated to construct the correlation matrix, and the soft threshold function is used to transform the correlation matrix into a weighted adjacency matrix. In order to obtain a balanced co-expressed network between scale independence and mean connectivity, a soft connectivity algorithm is used to calculate the scale independence and mean connectivity with different powers. We transform the adjacency matrix into topological overlap matrix (TOM). According to 1-tom as distance measurement, we classified gene clustering as co-expression modules, with a depth split value of 2, a minimum size cutoff value of 20, and a maximum module size of 5,000. To determine the association between co-expressed modules and clinical features, we cut the tree into different modules (minimum number of genes in a module is 30) using a dynamic shearing method by setting the soft threshold power to 1–10 and β = 16 (scale-free R^2 = 0.83) and cut the height to 0.3, using 0.25 as the merging threshold (shearing height), under which modules will be merged (correlation (modules with a correlation higher than 0.75 will be merged).

### GO and KEGG enrichment analysis

Gene ontology (GO) analysis and Kyoto Encyclopedia of Genes and Genomes (KEGG) pathway enrichment analysis were calculated using the R package Clusterprofiler program [24]. It includes annotation mining the function and path of the module, and identifying the dysfunctional module with function and path. Pvalue < 0.05 was considered significant, and the identified significant analyses were classified by gene counting.

### The analysis of immune cell infiltration

Cibersort is an online analysis tool for immunocell infiltration for a variety of diseases, including osteoarthritis, lupus nephritis, atopic dermatitis, acne and rosacea dermatitis [25–28]. At present, few studies have used the Cibersort method to study the characteristics of immune cell infiltration in tendinopathy. We assessed the proportion of immune cells in patients and the normal population by Cibersort analysis based on the genome-wide level of tendinopathy [29]. This revealed the characteristics of tendinopathy immune cell infiltration by heat mapping using ggplot2.

### Screening and identification of gene prediction model for early diagnosis

Lasso logistic regression is a machine learning method, which determines the variable by finding the λ value with the smallest classification error [30]. By processing the data of GSE26051 and GSE167226 processed, 75% of the samples in the data set are selected as the test set, 25% of the samples are used as the validation set, and the glmnet package of R is used as the binomial LASSO model of the training set. We plotted the operating characteristic curve of the recipient and determined its AUC. The diagnostic value of hub genes was evaluated using the pROC software package in R [31]. SVM-RFE is a machine learning method based on support vector machine, which finds the best variable by subtracting the feature vector generated by svm. We run the e1071 package to eliminate the recursive features of the difference genes obtained, and use the svmRFE function for data calculation. We set up folding by wrapping the entire feature selection and generalization error estimation process in the top loop of external cross-validation. Then, we use the Lapply function to perform feature ordering on all training sets. Finally, we obtain all folded top features through the top.features function, and use the loop function to estimate the generalization error of different numbers of top features using the 10x CV standard, so that the error rate reaches the lowest point, and finally the best gene signature is obtained. We used the above two algorithms to screen the hub genes of tendinopathy at the same time and obtained the same hub genes. Finally, the same hub genes were classified and screened by the Gaussian mixture model (GMMS). The Gaussian mixture model (GMMS) is a feasible screening method with better clustering performance [32]. Through repeated training of Gaussian mixture model algorithm, we screened the highest cluster in the optimal cluster in the figure, screened the optimal AUC value in the fifth category as 0.95, and identified the final candidate hub genes.

## Results

### Data preprocessing and the screening of differential genes

**Fig 1:**
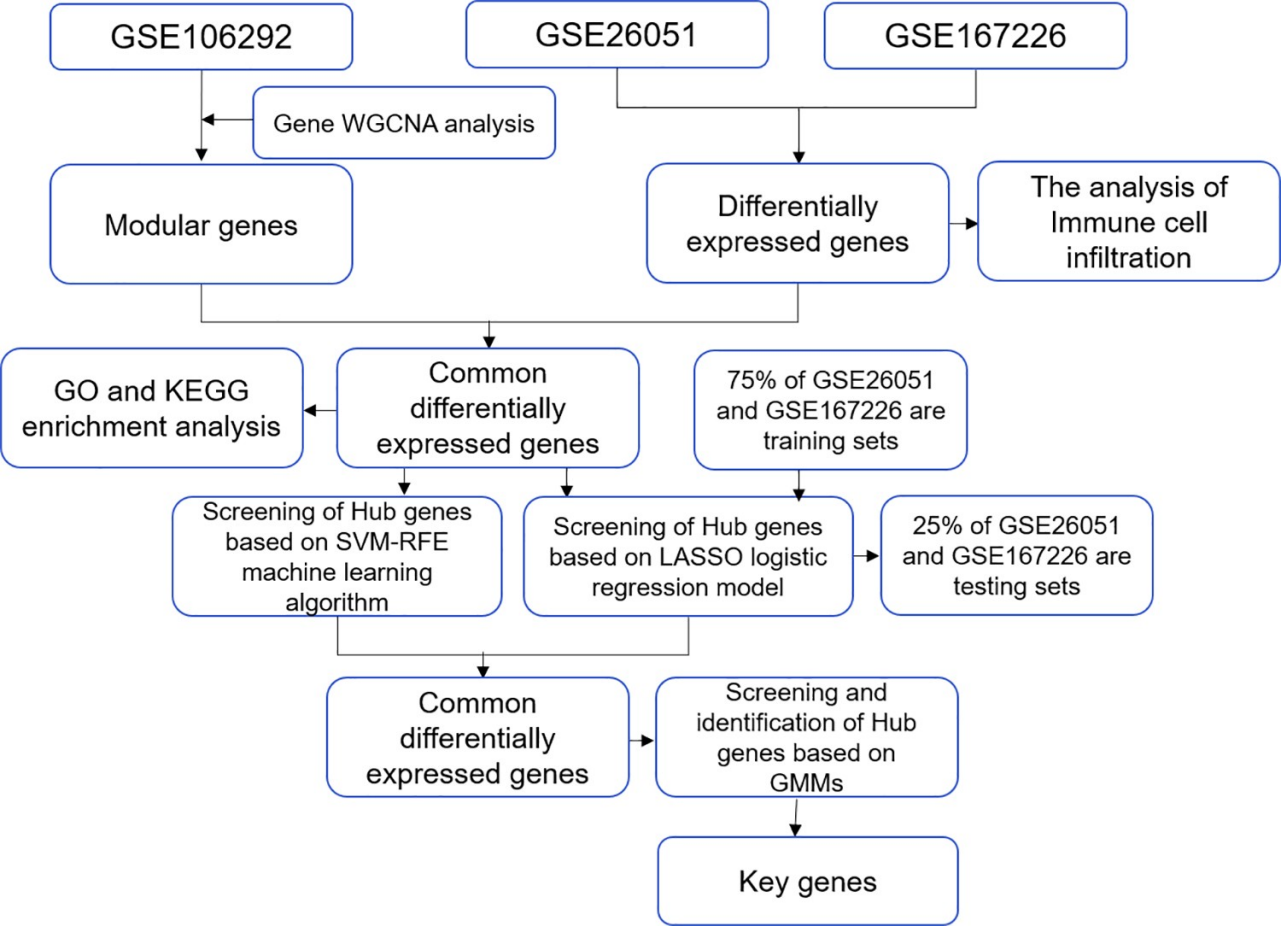
Flowchart of this study. The following datasets were used for the identification of potential diagnostic genes and mechanisms associated with the development of sepsis: GSE106292, GSE26051, GSE167226.

**Fig 2:**
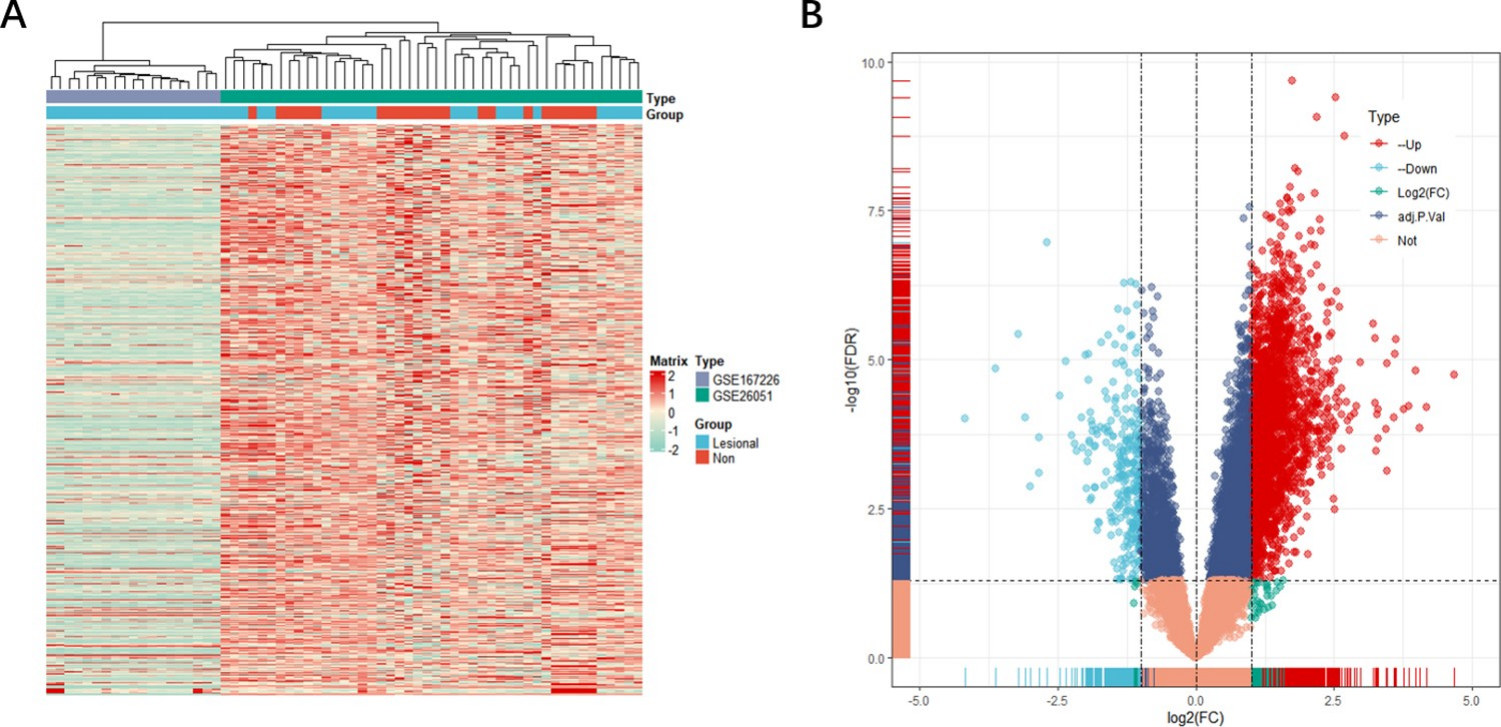
The gene differential expression analysis of GSE26051 and GSE167226 data set. (A) Whole gene expression heat map: Whole gene expression heat map of tendon tissue, with high expression in red and low expression in blue (B) The DEG Volcano map shows upregulated genes in red and down-regulated genes in blue.

The flow chart of this study is shown in Fig 1. After gene annotation and standardization of the data, GSE26051 data set contains 46 samples (2088 genes), and GSE167226 data set contains 19 samples (19402 genes). We showed by performing a whole-gene expression profile analysis of the combined data, Fig 2A shows the DEG expression heat map. Subsequent differential analysis and volcano mapping of counts genes (20,188 genes) in patient tissue and normal tissue samples were noted. A total of 2995 DEGs were identified, including 2729 up-regulated genes and 266 down-regulated genes (Fig 2B).

### Construction of weighted co-expression network and identification of key modules

**Fig 3:**
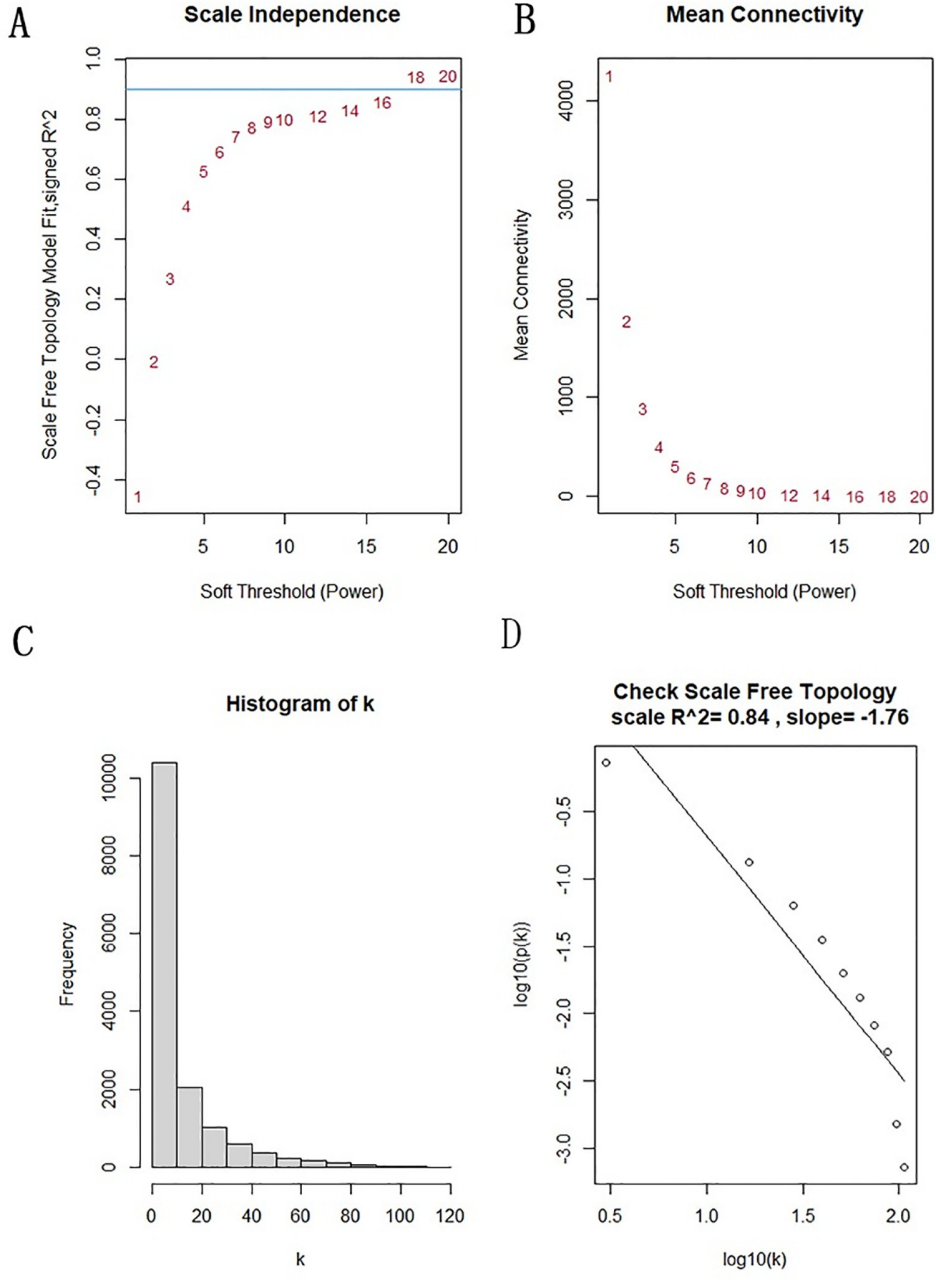
The screening criteria of WGCNA. (A)Soft Threshold (Power) represents the weight, and the vertical axis shows the scale-free topology fitting index R^2 (B) Soft Threshold (Power) represents the weight, and the vertical axis shows the average connectivity of the network (C) Distribution of node connectivity K (D) Correlation graph of K and P (K).

We performed WGCNA analysis on 20,226 genes that data set GSE106292 has been annotated. We preprocessed the sample expression values, and screened 15034 genes by screening standard deviation > 0.5. Based on the proximity matrix β = 16, we made the gene distribution conform to the scale-free network, sample tree and soft threshold estimation according to the connectivity degree. We set the vertical axis as the scale-free topology fitting index R^2 (the values in the SFT.R.Sq column in the statistical results) (Fig 3A) and the average connectivity of the network (Fig 3B). According to the negative correlation between k and p(k) (correlation coefficient 0.84), it can be shown that the selected β = 16 meets the standard of establishing the gene scale-free network (Fig 3C and 3D).

**Fig 4:**
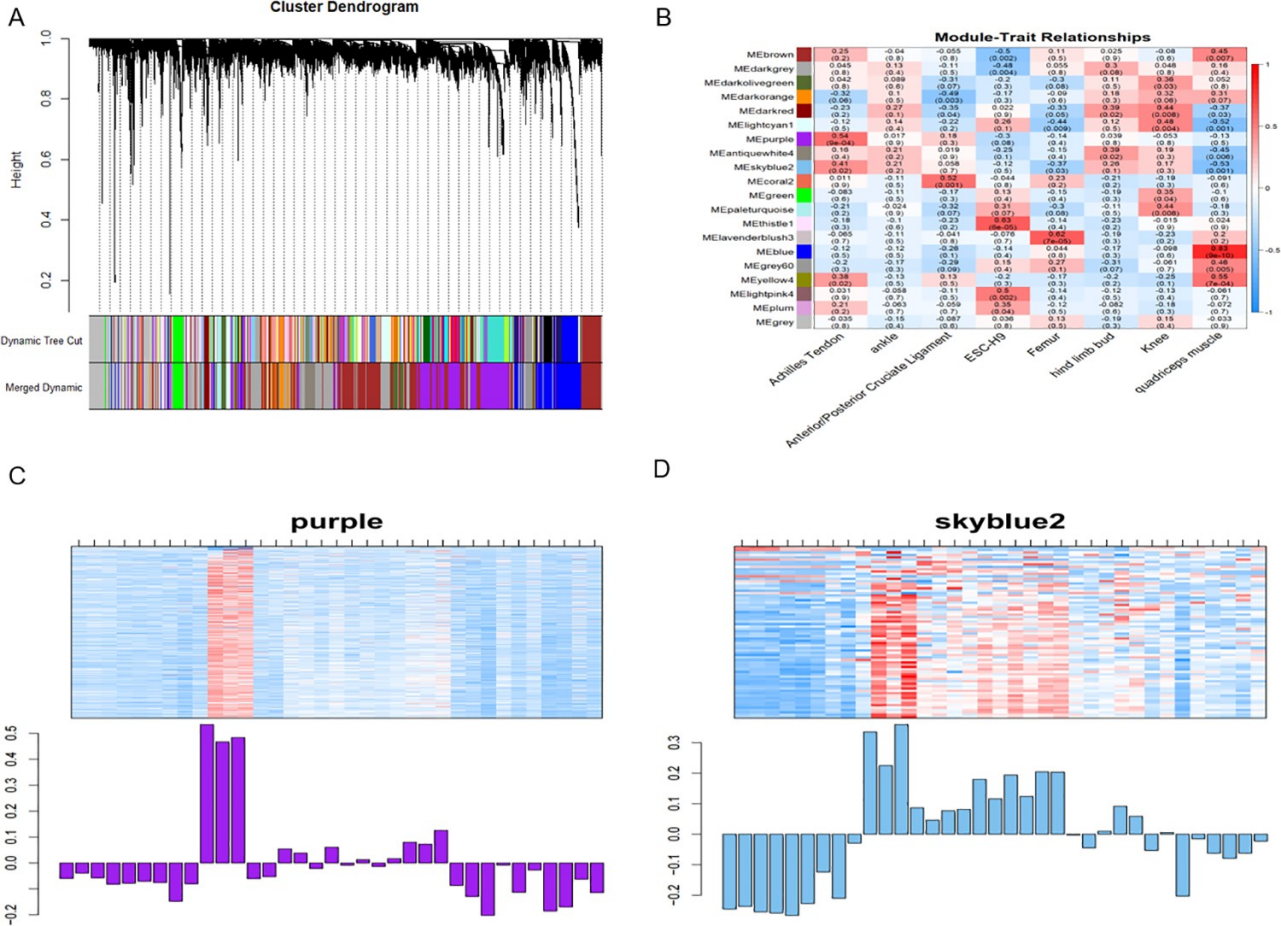
The WGCNA analysis of GSE106292 data set. (A)Tree of all gene expressions based on the Difference Measure (1-TOM) cluster (B)The heat maps of correlations between modules feature genes and samples, with each cell containing correlation coefficients and P values (C)The expression calorimetry and feature vector histogram of PURPRE module (D)The expression calorimetry and feature vector histogram of Skyblue2 module.

We set the soft threshold power to 1–10, β = 16 (without scale R^2 = 0.83) to obtain the final template as shown in Fig 4A. In the results of the respruning of the cluster tree, we identified 20 modules in the heat map of correlation between the tree diagram of gene expression and module features (Fig 4B). We selected the two modules with the highest correlation as the research target module, among which ModuleTraitCor in Purple = 0.54, ModuleTraitPvalue = 9E-04 and ModuleTraitCor in Skyblue2 = 0.41, ModuleTraitPvalue = 0.02 meets the criteria (Fig 4C and 4D).

### GO and KEGG enrichment analysis of differential genes

**Fig 5:**
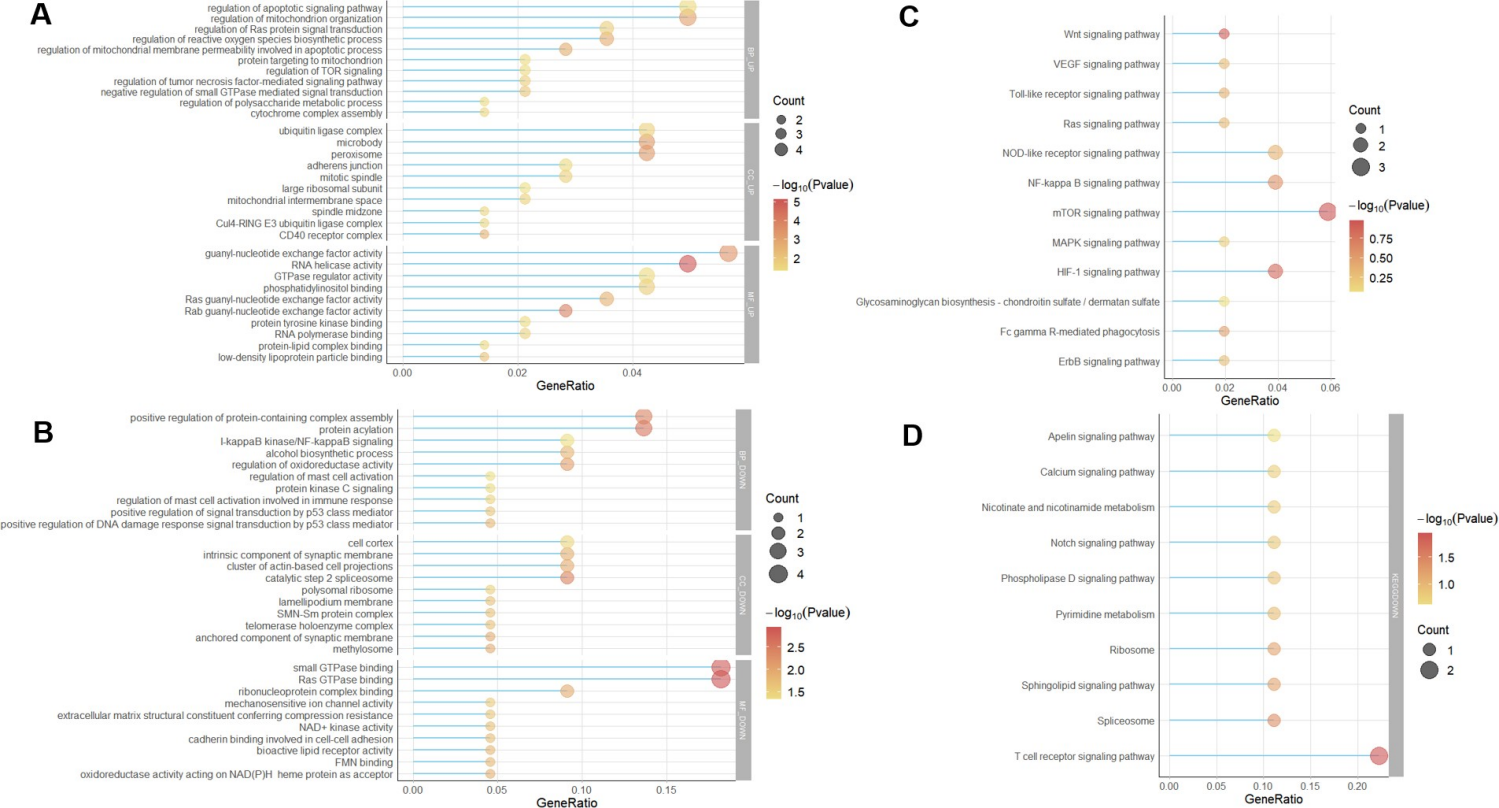
Baseball figure of differential gene enrichment analysis. The horizontal axis represents the proportion of differential genes in GO and KEGG enrichment analysis, and the vertical axis represents the enrichment category. (A)Up-regulated GO enrichment distribution of differentially expressed genes (B) Down-regulated GO enrichment distribution of differentially expressed genes (C) Up-regulated differential gene KEGG enrichment distribution (D) Down-regulated differential gene KEGG enrichment distribution.

A total of 171 genes were obtained from the intersection of genes and differential genes in the module, including 150 up-regulated genes and 21 down-regulated genes. GO and KEGG enrichment analysis were used for enrichment analysis of the obtained DEG using the clusterProfiler package of the R language. The results showed that the biological processes (BP) of the up-regulated genes were mainly related to the regulation of mitochondrion organization, the regulation of apoptotic signaling pathway, regulation of reactive oxygen species biosynthetic process, the regulation of tumor necrosis factor-mediated signaling pathway and other pathways. In terms of cell composition (CC), it is mainly related to peroxisome, microbody and ubiquitin ligase complex. In terms of molecular function (MF), it is mainly related to guanyl-nucleotide exchange factor activity, RNA helicase activity, GTPase regulator activity and phosphatidylinositol binding (Fig 5A and 5B). Among the down-regulated genes, biological processes (BP) are mainly related to protein acylation, positive regulation of protein-containing complex assembly, and I-kappaB kinase/NF-kappaB signaling. In terms of cell composition (CC), it is mainly related to catalytic step 2 spliceosome and a cluster of actin-based cell projections. In terms of molecular function (MF), it is mainly related to the binding of small GTPase binding and Ras GTPase binding (Fig 5A and 5B). The upregulated genes were mainly concentrated in mTOR, HIF-1, MAPK, NF-κB, NOD-like receptor and VEGF signaling pathways in the KEGG pathway rich concentration. The relatively down-regulated genes were mainly concentrated in T cell receptor signaling pathway, spliceosome, and sphingolipid signaling pathway (Fig 5C and 5D).

### The analysis of immune cell infiltration

**Fig 6:**
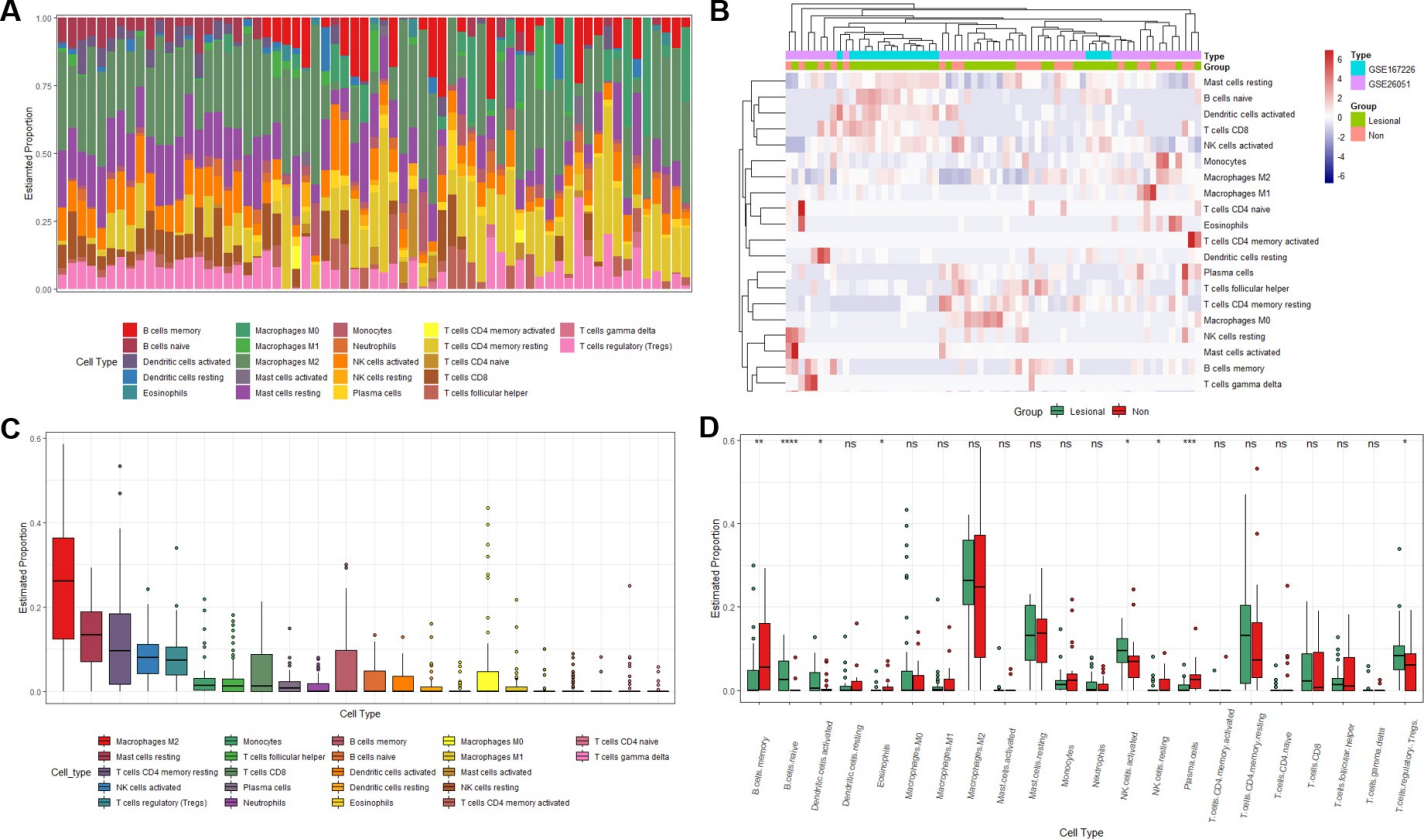
Infiltration patterns of immune cells in different groups. (A)Relative percentage of 22 immune cell subsets in tendon disease samples (B) Heat map of immune cell infiltration between tendinopathy group and control group, green represents tendinopathy group, red line represents control group (C) Infiltration degree of 22 immune cell subsets in tendon disease samples (D) Box Diagram of Immune Infiltration Difference between Tendon Disease Group and Control Group, Green as Tendon Disease Group, Red as Control Group.

We obtained the CIBERSORT absolute score for the analysis of immune cell infiltration based on the CIBERSORT algorithm, which can reflect the absolute content of 22 immune cells in each sample. According to CIBERSORT absolute score, we calculated the proportion of each infiltrating cell when the total infiltration rate was 100%. We mapped the overall proportion pattern of 22 subgroups of immune cells in tenopathy and healthy controls. The results showed that M2 macrophages, resting mast cells, neutrophils, activated NK cells and regulatory T cells (Tregs) were the highest infiltrating cells in all samples (Fig 6A). Heat maps of immune cell infiltration between the tendinopathy group and the control group showed significant enrichment of M1 macrophages, activated mast cells, γ-δT cells, regulatory T cells (Tregs), neutrophils, and M0 macrophages (Fig 6B). We found that M2 macrophages, resting memory CD4 + T cells, memory B cells, resting mast cells, CD8 + T cells, activated NK cells and regulatory T cells (Tregs) were highly infiltrated in all samples (Fig 6C). The differential expression between the two groups showed that the infiltration of regulatory T cells (Tregs), activated NK cells and naive B cells was higher than that of the control group (p < 0.05), while the infiltration of plasma cells and memory B cells in the tendon disease group was lower (p < 0.05) (Fig 6D).

### Screening and identification of hub genes in tendinopathy

**Fig 7:**
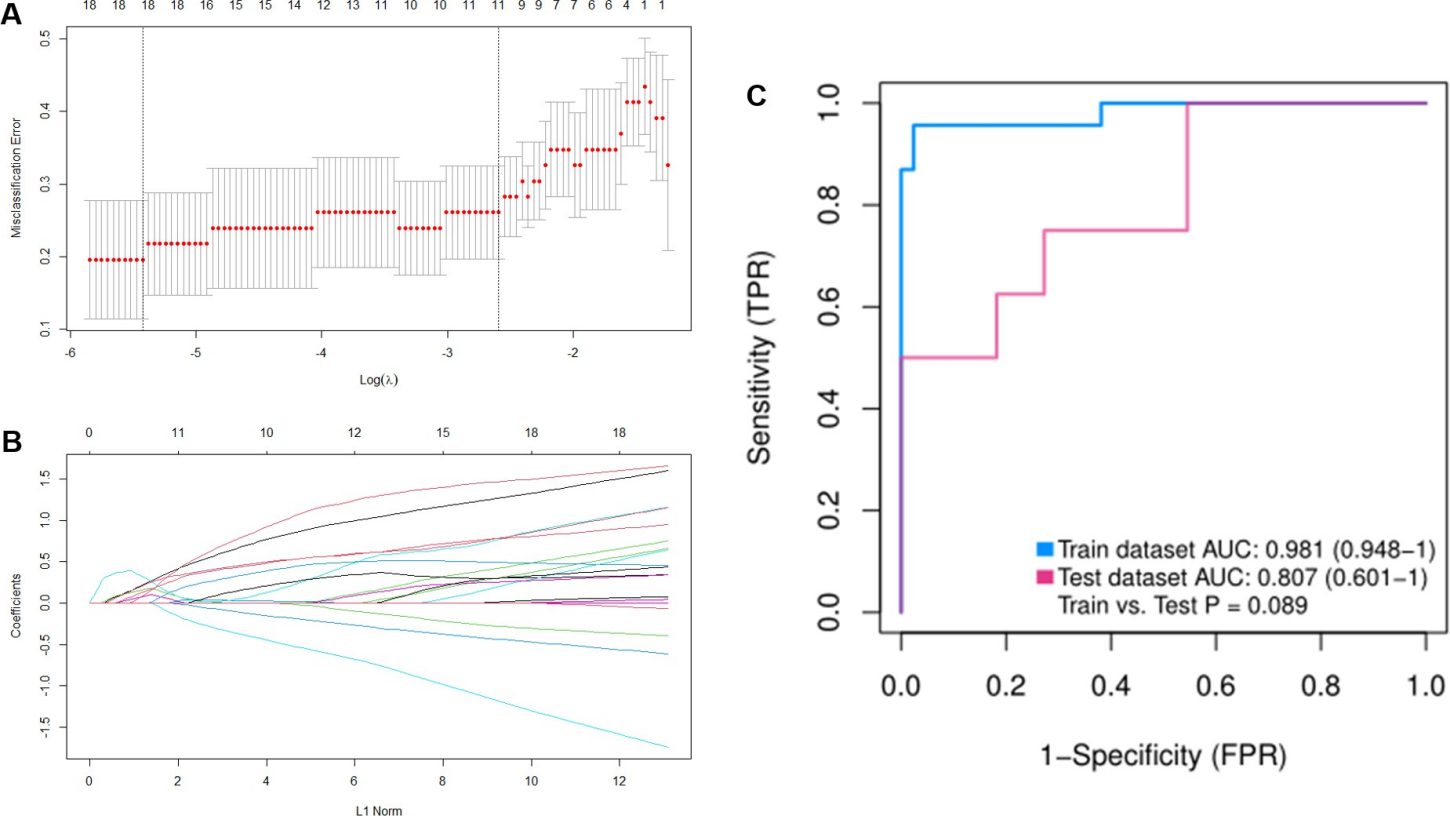
The potential key genes of tendinopathy were screened by LASSO regression model. In Fig 7A and 7B, the ordinate is the value of the coefficient, the lower abscissa is log(λ), and the upper abscissa is the number of non-zero coefficients in the model at this time. (A) Selection of the best parameter (number of non-zero coefficients in the model at this timet (B) LASSO coefficient spectrum of 18 differentially expressed genes selected by optimal (s timeti (C) Comparison of ROC curves between training set and validation set for gene signature.

First, we combine GSE26051 and GSE167226 data sets, and randomly divide all samples into a training set (75%) and verification set (25%). In order to prevent data snooping errors and avoid sample characteristics in the test set, we use the sample function in the R language to randomly use 80% of the data set as the training set and 20% as the test set. We implement model prediction through the predict function, that is, the fitCV object is the construction model, and the evaluation is performed in the two data sets of train and test. By constructing the LASSO model in the training set and selecting λ = 18 to determine the hub genes that can predict early tendinopathy accurately (Fig 7A). Based on the optimal λ value of 18, we obtained the LASSO coefficient spectrum of differentially expressed genes (Fig 7B). Normally, the training set is used to train the model, and the test set is used to evaluate the performance of the model as a whole. In this study, the AUC of the training set is 0.981>0.7, indicating that the built model has good real performance, and the AUC of the test set is 0.807>0.7, indicating that the built model has good generalization performance and excellent verification performance (Fig 7C). Finally, we obtained 18 genes with nonzero coefficients, including *MACROD1*, *CES2*, *GFER*, *MRPL52*, *SKIV2L*, *B3GNT4*, *LYNX1*, *C19orf57*, *SAFB2*, *NOM1*, *C7orf43*, *FRMPD1*, *MLPH*, *MFSD10*, *PIEZO1*, *FAM222A*, *PRG4*, *POU2AF1*.

**Fig 8:**
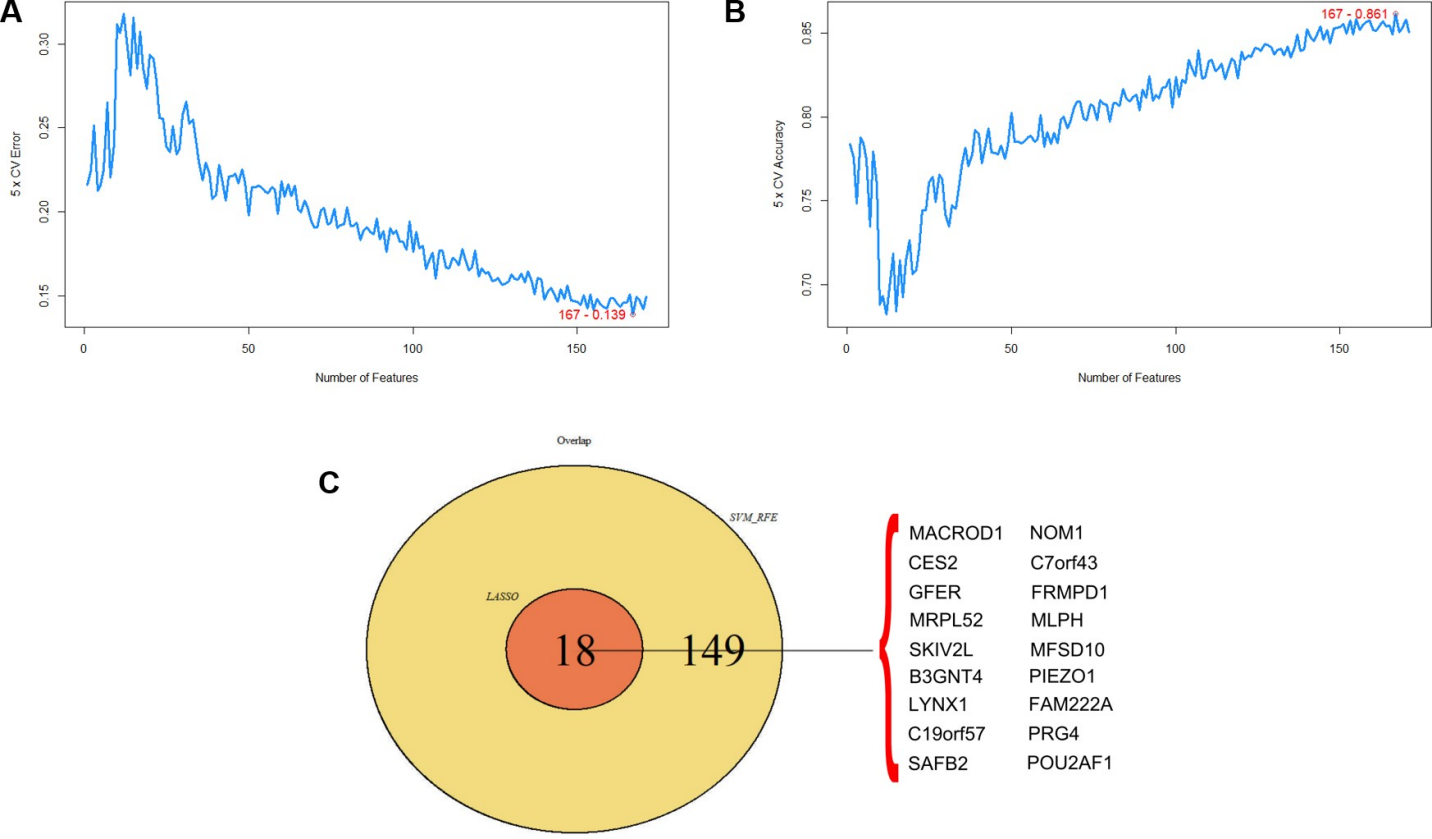
MSVM-RFE algorithm for screening key genes. (A)Shows the error rate of the SVM model (B) Shows the accuracy of the SVM model (C)The Venn diagram shows the same key genes obtained by the two algorithms.

At the same time, we used multiple support vector machine recursive feature elimination (mSVM-RFE) algorithm to screen 171 different genes, and obtained 167 hub genes. Build the SVM model by selecting the first 171 variables and check that the model error rate is 167–0.139, and the accuracy rate is 167–0.861. The position of the red circle is the lowest point of error rate (Fig 8A). The position of the red circle is the point with the highest accuracy (Fig 8B). We obtained common hub genes from the above two algorithms, including *MACROD1*, *CES2*, *GFER*, *MRPL52*, *SKIV2L*, *B3GNT4*, *LYNX1*, *C19orf57*, *SAFB2*, *NOM1*, *C7orf43*, *FRMPD1*, *MLPH*, *MFSD10*, *PIEZO1*, *FAM222A*, *PRG4*, *POU2AF1*(Fig 8C).

**Fig 9:**
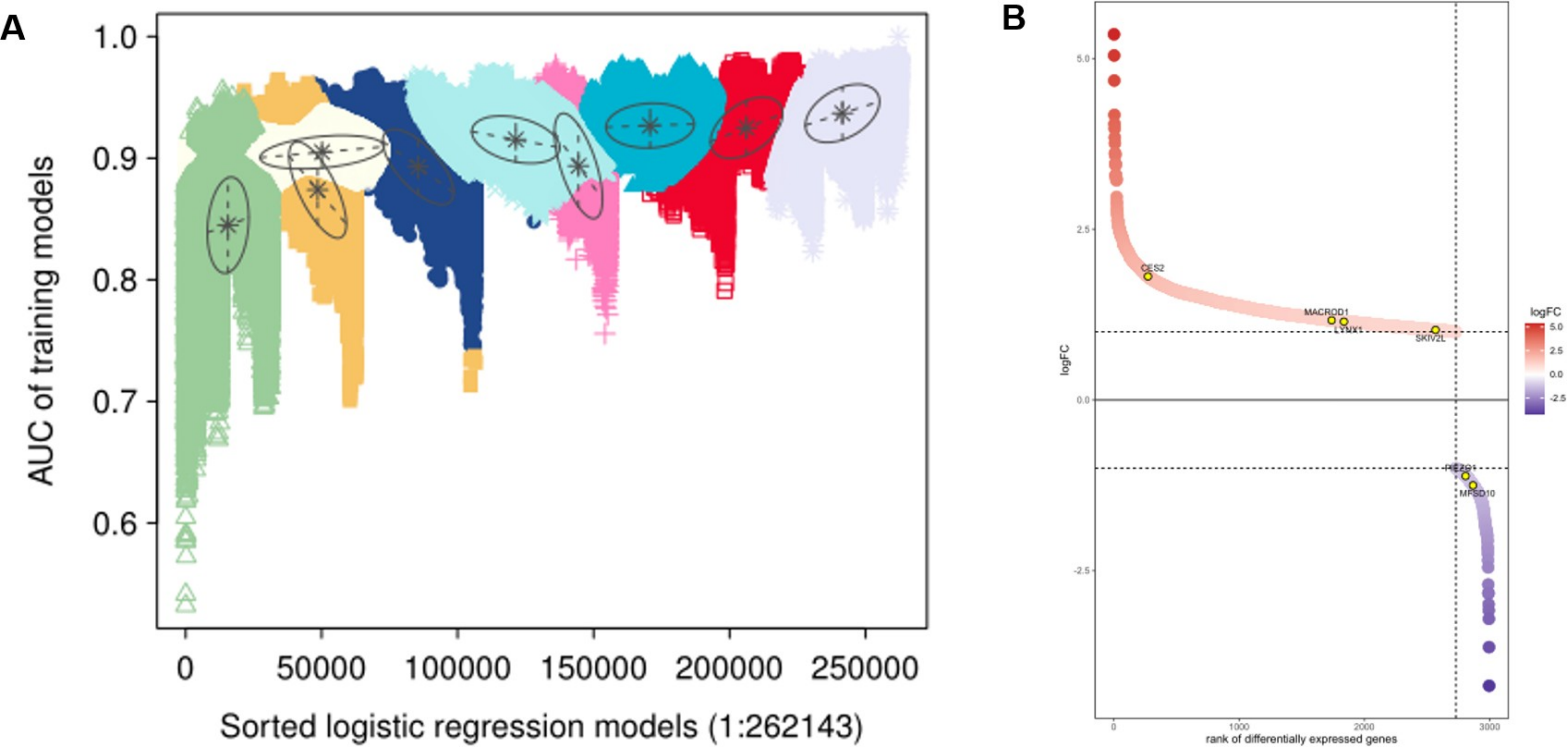
Displays the patterns of AUC and 262143 logistic regression model based on Gaussian finite mixture model. (A)The pattern of the logistic regression model is related to the AUC score and is determined by Gaussian mixture (B) The waterfall diagrams of 6 key genes in different genes.

In order to more accurately predict genes for the early diagnosis of tendinopathy. Based on the Gaussian finite mixture model, we use the model-based hierarchical agglomerative clustering method for classification. We use a Gaussian Mixture Model (GMM) to classify miRNA clusters, and use Logistic regression analysis to establish a combined model for predicting recurrence. At the same time, we constructed a receiver operating characteristic (ROC) curve, and calculated AUC to evaluate the predictive value of the model, using a predictive miRNA signature model. We used the Gaussian mixture model algorithm to calculate and verify the best hub genes from 18 candidate hub genes by constructing 262143 AUC models. Finally, we screened 6 potential hub genes, including *MACROD1*, *CES2*, *SKIV2L*, *LYNX1*, *MFSD10* and *PIEZO1* (Fig 9A). The FoldChange and p-value populations were used to display differential gene waterfall plots, and the predictive mRNA signature model was described using Gaussian finite mixture model markers (Fig 9B). The overall situation of the 6 potential hub genes is listed in Table 1.

**Table 1:** 6 potential hub gene display.

	logFC	AveExpr	t	P.Value	Type
**MACROD1**	1.167552501	5.712454732	5.41405282	8.56E-07	Up
**CES2**	1.809870576	7.075108564	5.355859588	1.07E-06	Up
**SKIV2L**	1.026460579	6.111613386	4.671443796	1.44E-05	Up
**LYNX1**	1.148012232	6.163685681	4.321936756	5.13E-05	Up
**MFSD10**	-1.250111926	5.106939598	-3.626082073	0.000548936	Down
**PIEZO1**	-1.112748319	6.687175452	-3.52261718	0.000765663	Down

## Discussion

Tendinopathy is not only a very common chronic disease, but also a disease that lacks real effective treatment [33]. So far, tendinopathy is still a major challenge in musculoskeletal diseases due to the widespread disease population, low cure rate, and huge medical expenditure. However, the mechanism of the occurrence and development of tendinopathy is not yet fully understood. At present, there are many hypotheses about the etiology of tendinopathy, including Biomechanical theory [34], inflammation theory [33], apoptosis theory [35], vascular or neurogenic theory [36], etc. Although these theoretical models closely link the basic science of tendinopathy with clinical applications, none of the theoretical theories can fully clarify the pathological mechanism of tendinopathy and the complex relationship between tendon pain and function. Although the current research on tendinopathy has involved many aspects, the hub genes of early tendinopathy are rarely studied. We believe that early diagnosis and treatment of tendinopathy is essential to prevent the further progression of tendinopathy to avoid the subsequent pathological cascade of tendinopathy. In this study, we finally screened out 6 key genes, including *MACROD1*, *CES2*, *SKIV2L*, *LYNX1*, *MFSD10*, and *PIEZO1*. Among them, the gene with the smallest PValue is *MACROD1*. We speculate that this gene may be important in the occurrence and development of tendinopathy.

The *MACROD1* gene is still rare in current research and its specific function is unclear. A few studies have proved the functions of *MACROD1* in the nucleus and the cytoplasm of the body, including *MACROD1* binding and regulating transcription factors ERα and NF-κB related proteins [37,38]. Recent studies have shown that endogenous *MACROD1* protein is highly enriched in mitochondria and is highly expressed in human and mouse skeletal muscle [39]. As we all know, mitochondria, as a kind of organelle, have abundant biological functions in cells [40]. Mitochondria are not only factories that produce ATP but also participate in many biological processes, such as steroid biosynthesis [41], metal ion homeostasis in the human body [42,43], immune cell activation and regulation [44], cell signaling [45], apoptosis [46] and inflammation [47]. At the same time, mitochondrial dysfunction can cause many diseases, such as atherosclerosis [48], Alzheimer’s disease [49]. Mitochondria can produce reactive oxygen species (ROS) in the process of oxidative phosphorylation. When the accumulation of ROS exceeds the cellular antioxidant defense system, the accumulation of ROS levels will cause oxidative stress. Oxidative stress can cause ROS-mediated damage to molecular substances such as proteins, nucleic acids, and lipids [50]. And some studies believe that oxidative stress is closely related to vascular disease [51], neurodegeneration [52], and cancer [53]. Similarly, ROS can also regulate the cascade reaction in the MAPK signaling pathway by activating apoptosis signal regulator 1 (ASK1) to induce apoptosis [54,55]. Therefore, based on the high enrichment of MacroD1 gene in mitochondria, high expression in skeletal muscle, and the biological function of mitochondria, we speculate that the occurrence of MacroD1 gene in early tendinopathy is related to the comprehensive cascade reaction of hypoxic microenvironment, inflammatory response, apoptosis and so on.

The *MACROD1* gene may trigger the hypoxic microenvironment-mediated tendon inflammation through mitochondrial dysfunction. As we all know, hypoxic cell injury has been considered as the basic mechanism of tendinopathy [56]. Research suggests that hypoxia may be a potential cause of early tendinopathy. Under the action of mechanical stimulation and injury, hypoxia promotes the release of inflammatory cytokines in human tendon cells, the expression of key apoptosis mediators, the formation of blood vessels [57], and significantly affects the synthesis of collagen matrix [58]. Previous research suggests that chronic tendinopathy is caused by a degenerative process without inflammation. In a study of biopsy components of tendinopathy rupture tissue, no obvious inflammation was observed, and more than 85% of the biopsy specimens had almost no inflammatory cells. On the contrary, there was a marked increase in tissue degeneration, including thinning and disorientation of collagen fibers, myxoid degeneration, hyaluronic acid degeneration, chondroid metaplasia, tissue calcification, angiogenesis, and fatty infiltration [59,60]. Interestingly, some recent studies have reached the opposite conclusion that there is an early and important inflammatory response in the process of tendinopathy. Molloy et al found that the expression of the inflammatory cell receptor and immunoglobulin was up-regulated in the rat supraspinatus tendon disease model by microarray analysis [61]. Matthews et al. showed that a small-area tear is more obvious in the inflammatory infiltration of macrophages and mast cells, reflecting more degenerative changes through a biopsy sample of tearing tendon tissue [62]. Interestingly, in our study, the KEGG enrichment pathway was significantly up-regulated by the NF-κB signaling pathway and the MAPK signaling pathway. Studies have shown that these two pathways are related to inflammation, apoptosis and ossification. For example, ERK regulates apoptosis, while P38 mediates apoptosis and inflammation [63]. And there are also studies suggesting that the activation of NF-κB can induce the activation of the MAPK pathway [64]. Another study showed that TNF-α can induce TDSC inflammation and apoptosis, and promote the development of tendinopathy by up-regulating the activation of MAPK and NF-κB pathways [65]. Our analysis of the difference in immune cell infiltration of the whole gene of the samples showed that the infiltration of M1 macrophages, activated mast cells, activated NK cells, and regulatory T cells (Tregs) in tendinopathy samples increased, while the infiltration of plasma cells and memory B cells decreased. Through a cross-sectional and case-control study, Maja et al. found that in chronic tendinopathy tissues, compared with healthy tendons, most (52%-96%) biopsy specimens were observed in macrophages, T lymphocytes, and hypertrophy Cells and natural killer cells [66]. This indicates that M1 macrophages, activated mast cells, and activated NK cells play an important role in the progression and treatment of tendinopathy. It can be considered that the *MACROD1* gene may induce inflammatory cell infiltration by mediating the hypoxic microenvironment.

Disregulation of apoptosis is thought to be one of the causes of tendinopathy [35]. Apoptosis is a kind of programmed cell death, which plays a key role in tissue homeostasis. Apoptosis causes many diseases, such as autoimmune diseases and skeletal muscle degeneration [67]. Current research believes that cell apoptosis are crucial in the occurrence and development of tendinopathy [35]. The reasons include: mechanical overuse of tendons [68], hypoxic microenvironment [69], and oxidative stress [70]. In our study, the differential gene enrichment pathway was significantly up-regulated on the HIF-1 signaling pathway. However, hypoxia-inducible factor 1 (HIF-1), as a transcriptional activator sensitive to oxygen [71], is a key regulator in the process of cell apoptosis [72]. In hypoxia, HIF-1α can initiate cell apoptosis by inducing high concentrations of pro-apoptotic proteins. Vascular endothelial growth factor (VEGF) is a glycosylated protein of about 45 kDa, composed of two subunits connected by disulfide bonds, which can mediate angiogenesis. Previous studies have suggested that VEGF is at a high level of expression in degenerative tendinopathy, while its expression is almost completely down-regulated in healthy Achilles tendons [73]. There are many factors that cause high expression of VEGF in tendon cells, including hypoxia, inflammatory factors and mechanical stress load [74]. As for the mechanism of action of VEGF in tendinopathy, some studies have shown that VEGF activates the binding of VEGF and its receptor VEGFR-2 to promote angiogenesis in tendon tissue by up-regulating the expression of matrix metalloproteinases (MMPs) and down-regulating the expression of metalloproteinase-3 (TIMP-3) in tendon cells [74–77]. Dakin et al. studied the tendon biopsy in symptomatic patients with tendinopathy or rupture and found that the superposition of inflammatory infiltration and neovascularization promotes tendon rupture [78]. Interestingly, in our study, based on KEGG enrichment, the VEGF signaling pathway is significantly up-regulated, which may lead us to speculate that VEGF plays an important role in the early pathogenesis of tendinopathy under hypoxic factors.

In the development of tendinopathy, the wrong differentiation of tendon cells may promote the occurrence of tendinopathy [79]. Hypoxia and inflammation are related to the occurrence of heterotopic endochondral ossification (HEO), but the specific molecular mechanism is unclear. It has been proven that hypoxic environment can stimulate the differentiation of progenitor cells into cartilage during the development of the skeletal system [80]. Wang et al. believed that cell hypoxia promoted heterotopic ossification by amplifying BMP signal transduction [81]. Shailesh et al. in the rat Achilles tendon, muscle ossification model and FOP mouse model found that HIF-1α was significantly up-regulated in the chondrogenic differentiation stage [82]. Tendon-derived stem cells (TDSC) have the potential to differentiate into tendon cells, osteoblasts, chondrocytes and fibroblasts [83]. TDSC plays an important role in the healing of tendon injuries. However, if the tendon is not properly healed, it will cause tendon ossification and promote the formation of tendinopathy [84–86]. Studies have shown that TNF-α can induce apoptosis of TDSC [87], and chronic tendinopathy is closely related to the up-regulation of TNF-α [88]. This is closely related to our study and screening of different genes in the regulation of tumor necrosis factor-mediated signaling pathways on GO enrichment. Some studies believe that Wnt pathway mediators are expressed in chondrocyte-like cells and ossification deposits, and are related to endochondral ossification [89]. Differentiation of tendon stem cells into non-tendon cells, such as osteoblasts, may reduce the total amount of tendon stem cells used for tendon repair and lead to failure of tendon healing. Studies have shown that activation of the Wnt pathway can promote calcification of related tissues, such as cardiovascular calcification [90,91]. In our study, the Wnt pathway was significantly up-regulated. We believe that the Wnt pathway is activated and at a high level of expression in tendinopathy injuries. And Wnt induces the osteogenic differentiation of tendon stem cells, and promotes the differentiation of tendon cells in the wrong direction, which ultimately leads to tendinopathy [92]. Studies have shown that in the rat Achilles tendon injury model, the key regulators of the Wnt pathway and the Notch pathway are activated at the wound [93]. Therefore, it can be considered that the *MACROD1* gene may promote the misdifferentiation of tendon cells by mediating the hypoxic microenvironment of the cells and ultimately lead to tendinopathy.

Based on the above research, we speculate that the up-regulation of *MACROD1* gene may cause early tendinopathy hypoxia microenvironment and oxidative stress response through tendinocyte mitochondrial dysfunction, and then activate multiple signal pathways under the combined action of inflammatory cytokines and angiogenic factors Lead to apoptosis of tendon cells. These cascades ultimately lead to the development of chronic tendinopathy. On the other hand, we used the online tool CIBERSORT algorithm to analyze the immune cell infiltration to get the difference in the content of immune cell infiltration in 22. This may have important implications for tendinopathy in the study of immune cells. In short, we speculate that the *MACROD1* gene may be a potential hub gene for early tendinopathy. It is necessary for us to study clearly the function of *MACROD1* gene in mitochondria and the possible specific molecular mechanism of the early occurrence of tendinopathy. By exploring the mechanism of *MACROD1* gene in mitochondria and the characteristics of immune cell infiltration, we can find new therapeutic targets in molecular pathways, which may be a promising treatment method for tendinopathy.

In recent decades, computational models have become an important tool for the identification of novel MicroRNAs, LncRNAs and CircRNAs in association with diseases. Meanwhile, a large number of experimental methods and computational models have been designed and implemented to identify novel MicroRNA, LncRNA, and CircRNA associations with complex diseases, which contribute to the understanding of human complex disease mechanisms, biomarker detection, and disease diagnosis, treatment, prognosis, and prevention at the molecular level [94–97]. And, there are also studies on LncRNA-MicroRNA interaction prediction by network distance analysis model, which has an important role in the screening of therapeutic targets and diagnostic biomarkers for a variety of human diseases [97–99]. On the other hand, logistic matrix factorization with neighborhood regularized (LMFNRLMI) is a new matrix factorization model for predicting the interaction of lncRNA-miRNA. Research shows that through comparison with several other network algorithms and various similarity tests, the model is superior and has higher performance in predicting the association of lncRNA-miRNA [100]. In the future, we can use LMFNRLMI to predict potential lncRNA-miRNA association studies in tendinopathy. Unfortunately, association studies at the transcriptome level with tendinopathies using bioinformatics approaches have been inadequate in recent years. Therefore, more research is needed in the future on top of computational modeling studies of tendinopathy gene biomarker identification and association studies at the transcriptome level.

However, our research still has certain limitations. First of all, the data set we are studying contains different populations of tendon tear patients and the control group, which may affect the results of the study. In addition, there is a slight difference between the immune cell infiltration condition obtained by the whole genetic immunoassay and the immune cell infiltration condition obtained from the experimental study, which may be caused by the difference in different stages of the disease. Finally, based on our research data comes from public databases, it is necessary to conduct molecular cell and animal experiments to verify the results of this research.

## Conclusion

In conclusion, based on the comprehensive bioinformatics analysis method, we identified the potential early hub genes, key regulatory pathways and immune infiltration characteristics of tenopathy. This will help to provide new insights into the future drug and molecular mechanism of tendon disease.
